# Multifocal high-grade glioma radiotherapy safety and efficacy

**DOI:** 10.1186/s13014-021-01886-3

**Published:** 2021-08-28

**Authors:** Daniel Felix Fleischmann, Rudolph Schön, Stefanie Corradini, Raphael Bodensohn, Indrawati Hadi, Jan Hofmaier, Robert Forbrig, Niklas Thon, Mario Dorostkar, Claus Belka, Maximilian Niyazi

**Affiliations:** 1 Department of Radiation Oncology, University Hospital, LMU Munich, Munich, Germany; 2 German Cancer Consortium (DKTK), partner site, Munich, Germany; 3 German Cancer Research Center (DKFZ), Heidelberg, Germany; 4 Institute of Neuroradiology, University Hospital, LMU Munich, Munich, Germany; 5 Department of Neurosurgery, University Hospital, LMU Munich, Munich, Germany; 6 Institute of Neuropathology, Faculty of Medicine, LMU Munich, Munich, Germany

**Keywords:** Multifocal high-grade glioma, Radiotherapy, Safety, Efficacy

## Abstract

**Background:**

Multifocal manifestation of high-grade glioma is a rare disease with very unfavourable prognosis. The pathogenesis of multifocal glioma and pathophysiological differences to unifocal glioma are not fully understood. The optimal treatment of patients suffering from multifocal high-grade glioma is not defined in the current guidelines, therefore individual case series may be helpful as guidance for clinical decision-making.

**Methods:**

Patients with multifocal high-grade glioma treated with conventionally fractionated radiation therapy (RT) in our institution with or without concomitant chemotherapy between April 2011 and April 2019 were retrospectively analysed. Multifocality was neuroradiologically assessed and defined as at least two independent contrast-enhancing foci in the MRI T1 contrast-enhanced sequence. IDH mutational status and MGMT methylation status were assessed from histopathology records. GTV, PTV as well as the V30Gy, V45Gy and D2% volumes of the brain were analysed. Overall and progression-free survival were calculated from the diagnosis until death and from start of radiation therapy until diagnosis of progression of disease in MRI for all patients.

**Results:**

20 multifocal glioma cases (18 IDH wild-type glioblastoma cases, one diffuse astrocytic glioma, IDH wild-type case with molecular features of glioblastoma and one anaplastic astrocytoma, IDH wild-type case) were included into the analysis. Resection was performed in two cases and stereotactic biopsy only in 18 cases before the start of radiation therapy. At the start of radiation therapy patients were 61 years old in median (range 42–84 years). Histopathological examination showed IDH wild-type in all cases and MGMT promotor methylation in 11 cases (55%). Prescription schedules were 60 Gy (2 Gy × 30), 59.4 Gy (1.8 Gy × 33), 55 Gy (2.2 Gy × 25) and 50 Gy (2.5 Gy × 20) in 15, three, one and one cases, respectively. Concomitant temozolomide chemotherapy was applied in 16 cases, combined temozolomide/lomustine chemotherapy was applied in one case and concomitant bevacizumab therapy in one case. Median number of GTVs was three. Median volume of the sum of the GTVs was 26 cm^3^. Median volume of the PTV was 425.7 cm^3^ and median PTV to brain ratio 32.8 percent. Median D2% of the brain was 61.5 Gy (range 51.2–62.7) and median V30Gy and V45 of the brain were 59.9 percent (range 33–79.7) and 40.7 percent (range 14.9–64.1), respectively. Median survival was eight months (95% KI 3.6–12.4 months) and median progression free survival after initiation of RT five months (95% CI 2.8–7.2 months). Grade 2 toxicities were detected in eight cases and grade 3 toxicities in four cases consisting of increasing edema in three cases and one new-onset seizure. One grade 4 toxicity was detected, which was febrile neutropenia related to concomitant chemotherapy.

**Conclusion:**

Conventionally fractionated RT with concomitant chemotherapy could safely be applied in multifocal high-grade glioma in this case series despite large irradiation treatment fields.

## Introduction

Multifocal high-grade glioma is a primary brain tumour with the most unfavourable prognosis. Median overall survival times are still reported as low as eight months in median, despite aggressive treatment [1]. While the current World Health Organisation (WHO) classification does not refer to multifocal high-grade glioma as a specific subentity [2], multifocal high-grade glioma has been described as being molecular distinct from unifocal high-grade glioma in several histopathological studies [3–6].

Practice changing studies on the treatment of high-grade glioma have included patients with multifocal tumours, but did not analyse the prognosis and therapeutic outcomes of this subset of patients in detail [7–9]. In current guidelines, the therapeutic management of patients with multifocal high-grade glioma is therefore not defined separately from the treatment of unifocal high-grade gliomas [10–12]. Recommendations on the best treatment of multifocal high-grade glioma patients are still limited to institutional case series and database analyses.

Case series and database analyses of the radiation therapy (RT) treatment of multifocal high-grade glioma patients have focused on different fractionation regimes comparing conventionally fractionated with hypofractionated radiotherapy, as well as on the use of concomitant chemotherapy leading to differing recommendations [1, 13–17]. Unfortunately, multifocal high-grade glioma has been defined differently in many retrospective case series and a multitude of different treatment regimens with only limited information about related adverse events have been reported, which limits the comparability of these analyses. In particular, older case series without high-resolution MRI and state-of-the-art RT treatment techniques have limited transferability to the current treatment of patients with multifocal high-grade gliomas.

The aim of the present case series was to evaluate the RT treatment planning parameters, adverse events and the treatment outcome of modern high-precision RT with or without concomitant chemotherapy in order to better understand and improve the treatment of multifocal high-grade glioma patients.

## Methods

### Patients

Patients with primary diagnosis of a multifocal high-grade glioma, who underwent RT at our department between April 2011 and April 2019 were retrospectively analysed.

### Histopathologic examination

Histopathological confirmation of high-grade glioma in tissue samples obtained by stereotactic biopsy or neurosurgical resection was available for all patients. Mutation of the IDH1 gen and the IDH2 gen and MGMT promotor methylation status at the time of diagnosis were also available for all patients included in this retrospective analysis.

### Magnetic resonance imaging and definition of multifocal gliomas

MRI with contrast-enhanced T1 and T2 or FLAIR sequences were conducted prior to RT for all patients. Only patients with multifocal growth pattern at the time of first diagnosis as assessed by an experienced neuroradiologist were included in the study. High-grade gliomas were defined as multifocal, which comprised at least two independent contrast-enhancing foci in the MRI T1 contrast-enhanced sequence.

### Radiotherapy protocols

The indication for RT was based on the consensus recommendation of the interdisciplinary neuro-oncology tumour board in all cases. All patients were treated with limited field irradiation. Prior to radiotherapy, an individual thermoplastic mask was individually made for each patient to ensure reproducibility of patient positioning during planning CT and the following course of irradiation. The planning CT scan was performed with slice thickness of 3 mm.

**Fig. 1:**
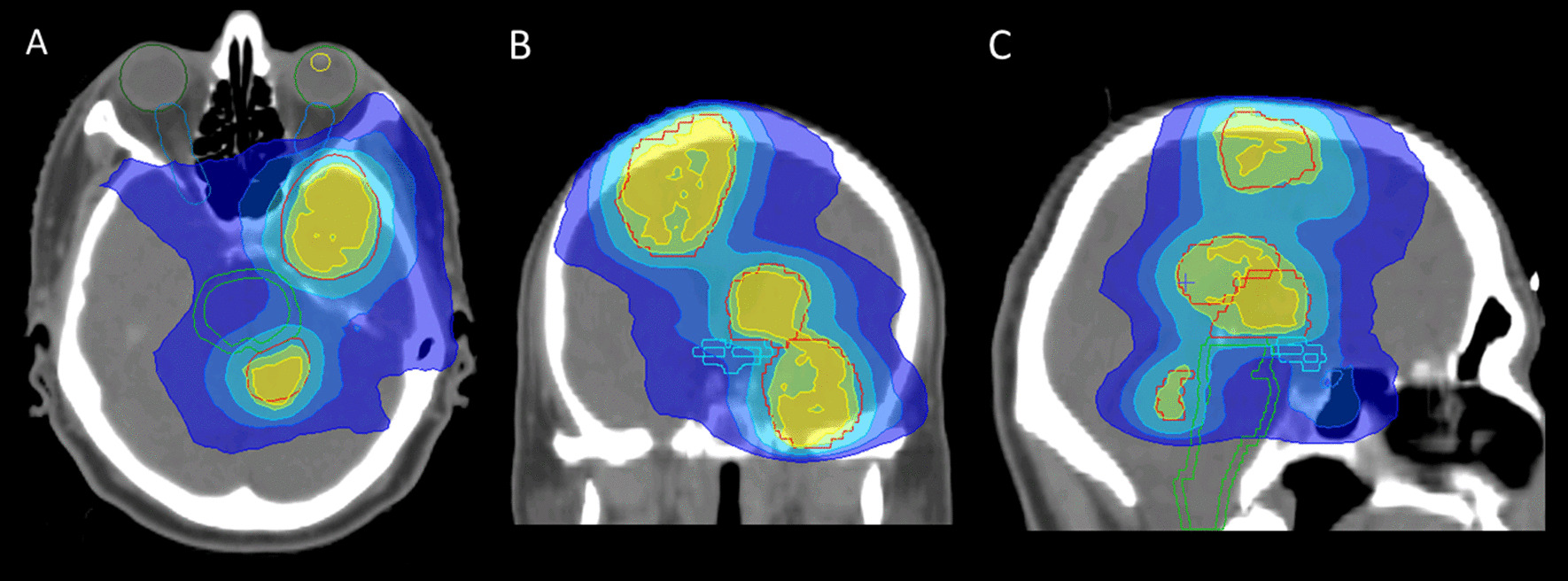
VMAT plan example. 69 year old male patient with multifocal glioblastoma treated at 5 foci with 60 Gy (2 Gy × 30) and concomitant temozolomide chemotherapy. Axial (**A**), coronal (**B**) and sagittal (C) plane of VMAT plan with PTV (red), 60 Gy isodose line (yellow), 57 Gy isodose line (green), 45 Gy isodose line (light blue), 35 Gy isodose line (blue) and 25 Gy isodose line (dark blue)

Radiation treatment plans included 3D conformal, intensity-modulated radiation therapy (IMRT) and volumetric modulated arc therapy (VMAT) plans (Fig. 1). Four different irradiation regimens were administered: 60 Gy (2 Gy × 30), 59.4 Gy (1.8 Gy × 33), 45 Gy (1.8 Gy × 25) with a simultaneous integrated boost (SIB) of 55 Gy (2.2 Gy × 25) and 40 Gy (2 Gy × 20) with a SIB of 50 Gy (2.5 Gy × 20). Contrast-enhanced T1 sequences, T2 and/or FLAIR sequences of MRI were co-registered with the planning CT imagines within the Oncentra External Beam® treatment planning system (version 4.5.2, Nucletron, 3905 TH Veenendaal, Netherlands).

Concomitant and adjuvant chemotherapy was administered according to the protocol of the EORTC 26,981/22981 NCIC CE.3 trial [7], with temozolomide and lomustine according to the protocol of the CeTeG/NOA-09 trial [18] or with bevacizumab with 10 mg per kilogram bodyweight every other week in analogy to Gutin et al. [19].

### Target volumes

The gross tumour volume (GTV) included all contrast-enhancing regions detected on T1-weighted MRI. For patients treated with 60 Gy, the clinical target volume (CTV) was based on the GTV with a margin of up to 20 mm and the perifocal edema visible on the T2 or FLAIR sequence accounting for microscopic tumour spread. The planning target volume (PTV) was created by anatomical adaptation of the corresponding CTV and a subsequent addition of a 3 to 5 mm margin to compensate for possible deviations in patient positioning. For patients treated with 59.4 Gy, the treatment volume receiving 50.4 Gy was created using the GTV with a 20 mm CTV margin and a 5 mm PTV margin and the boost volume receiving additional 9 Gy on a 10 mm GTV to PTV margin. For the patient treated with 45 Gy and a 55 Gy SIB, the 45 Gy volume was created on basis of the GTV with a 20 mm CTV margin expansion and a 3 mm PTV margin expansion and the SIB volume of 55 Gy on basis of a 10 mm GTV to PTV boost margin. In the patient treated with 40 Gy and a 50 Gy SIB, the 40 Gy volume was created on basis of the GTV with a 15 mm CTV margin expansion and a 3 mm PTV margin expansion and the SIB volume of 50 Gy on basis of a 5 mm GTV to PTV boost margin. For each case the overall GTV volume was calculated as the sum of all contrast-enhancing foci.

### Dosimetrical parameters

The dosimetrical parameters of the mean dose of the brain, the V30 and V45 and the D2 of the brain were assessed. Parameters V30 and V45 indicate the percentage of total brain tissue exposed to at least 30 Gy and 45 Gy, respectively. D2 describes the radiation exposure of the 2% brain tissue with the highest radiation exposure, measured in Gy.

### Statistical and survival analysis

Statistical analysis was performed with IBM^©^ SPSS^©^ Statistics (version 26, IBM^©^, Armonk, NY, USA). Descriptive statistical analysis was performed for patient characteristics, radiotherapy and dosimetric parameters. Kaplan–Meier estimator analyses were performed for overall and progression-free survival. Overall survival was calculated as the time interval between histological confirmation and the date of death or loss to follow-up. Progression-free survival was assessed as the time interval between initiation of RT and the first imaging detection of progressive disease according to the RANO criteria [20] or loss to follow-up.

### Assessment of adverse events

Adverse events, that occurred during or after the radiation treatment and which could have been linked to it, were evaluated and classified following the CTCAE 5.0 classification system.

## Results

### Patients

20 patients with multifocal high-grade glioma were examined, of which seven were female and 13 were male. The median age of all patients was 61 years (range 42–84 years). Median KPS prior to radiotherapy was 85 (range 50–100) and median KPS at the end of the radiotherapy was 80 (range 50–100). Patient characteristics for all patients are shown in Table 1.

**Table 1:** Patient characteristics

Pat	Age	Sex	Diag	WHO	IDH	MGMT	Resection	KPS pre RT	KPS post RT
1	50	Male	GB	IV	IDH WT	unmeth	biopsy	70	70
2	84	Male	GB	IV	IDH WT	meth	biopsy	80	80
3	68	Male	GB	IV	IDH WT	meth	biopsy	80	70
4	70	Male	GB	IV	IDH WT	meth	biopsy	90	90
5	74	Female	GB	IV	IDH WT	meth	biopsy	60	50
6	62	Male	GB	IV	IDH WT	unmeth	biopsy	90	80
7	63	Male	GB	IV	IDH WT	meth	biopsy	90	80
8	50	Female	GB	IV	IDH WT	unmeth	biopsy	90	80
9	68	Male	GB	IV	IDH WT	unmeth	biopsy	60	50
10	64	Female	GB	IV	IDH WT	unmeth	biopsy	80	70
11	53	Male	GB	IV	IDH WT	unmeth	str	90	80
12	50	Male	GB	IV	IDH WT	unmeth	biopsy	90	80
13	57	Female	AA	III	IDH WT	meth	biopsy	90	70
14	42	Female	GB	IV	IDH WT	meth	biopsy	80	70
15	62	Male	GB	IV	IDH WT	meth	gtr	100	100
16	50	Female	GB	IV	IDH WT	meth	biopsy	50	50
17	57	Male	GB	IV	IDH WT	meth	biopsy	100	100
18	54	Female	GB	IV	IDH WT	meth	biopsy	70	70
19	60	Male	GB	IV	IDH WT	unmeth	biopsy	70	80
20	69	Male	GB	IV	IDH WT	unmeth	biopsy	100	90

### Histopathological diagnosis

All patients underwent neurosurgical intervention prior to radiotherapy. 18 patients underwent stereotactic biopsy, while a resection was performed in two patients. Histological and molecular genetic examination of the collected tissue samples resulted in 18 IDH wild-type glioblastoma cases, one diffuse astrocytic glioma, IDH wild-type case with molecular features of glioblastoma and one anaplastic astrocytoma, IDH wild-type case. Examination of mutation of the IDH1 and IDH2 gene showed IDH1 and IDH2 wildtype in all cases. Examination of the MGMT promotor methylation status showed methylation of the MGMT promotor in 11 of 20 patients (55%).

### Treatment

Regarding radiotherapy prescription, 15 patients received 60 Gy (2 Gy × 30), three patients 59.4 Gy (1.8 Gy × 33), one patient 55 Gy (2.2 Gy × 25) and one patient 50 Gy (2.5 Gy × 20). Concomitant chemotherapy was administered in 18 patients. 16 patients were treated with temozolomide according to the protocol of the EORTC 26,981/22981 NCIC CE.3 trial, one patient with temozolomide and lomustine according to the CeTeG/NOA-09 trial and one patient with bevacizumab with 10 mg per kilogram bodyweight every other week.

Adjuvant chemotherapy was administered with temozolomide according to the protocol of the EORTC 26,981/22981 NCIC CE.3 trial in nine patients, with temozolomide and lomustine according to the CeTeG/NOA-09 trial in two patients and in one patient with bevacizumab with 10 mg per kilogram bodyweight every other week.

Treatment at progression was best supportive care in 15 cases, combined bevacizumab and irinotecan treatment in two cases, reRT with concomitant temozolomide chemotherapy with 36 Gy (2 Gy × 18) in one case and temozolomide rechallange at first progression and with reRT with 39 Gy (3 Gy × 13) within the GLIAA protocol at second progression in one case [21]. In one case, there was no progression at the time of data analysis.

### Target and dosimetrical volumes

Average number of GTVs was three GTVs with a range from two GTVs up to nine GTVs. The median size of the sum of the GTVs was 26 cm^3^ (range 3.6–303.9 cm^3^). The examination of the dosimetrical parameters showed a median D2% of 61.5 Gy. The percentage proportion of V30Gy and V45Gy was 59.9% (range 33–79.7%) and 40.7% (range 14.9–64.1%), respectively. The median percentage of the high dose irradiated volume divided by the brain volume was 32.8 percent (range 12–63.2%). Radiotherapy and dosimetric parameters for all patients are shown in Table 2.

**Table 2:** Radiotherapy and dosimetric parameters

Pat	RT Plan	Dose (Gy)	Conc. Ctx	Adj. Ctx	GTV Sum (ccm)	PTV (ccm)	Brain (ccm)	PTV/brain ratio (%)	Mean brain dose (Gy)	D2 Brain (Gy)	V30 Brain (%)	V45 Brain (%)	Treatment at progression
1	3D CRT	60	TMZ	TMZ	67.2	654.1	1554.9	42.1	44.3	61.5	74.1	64.1	BSC
2	3D CRT	60	TMZ	TMZ	20.1	446.8	1252.7	35.7	37.9	61.5	62.3	53.5	BSC
3	3D CRT	59.4	TMZ	none	48.6	573.8	1464.5	39.2	38.3	60.2	68.4	53.5	BSC
4	3D CRT	59.4	TMZ	TMZ	39.8	544.3	1435.1	37.9	37.4	62.1	58.3	49	BSC
5	3D CRT	59.4	TMZ	none	10	567.3	1179	48.1	37.6	60.1	61.6	48.7	BSC
6	3D CRT	60	TMZ	TMZ	16.7	435.5	1388.8	31.4	37.2	61.3	54.4	41.8	BSC
7	IMRT	60	TMZ	TMZ	58	833.8	1466.1	56.9	43	60.7	79.3	53.8	BSC
8	IMRT	60	TMZ	none	113.6	578.3	1372.5	42.1	42.5	62.7	68.4	46.7	BEV/IRI
9	IMRT	60	TMZ	TMZ	21.9	415.9	1410.9	29.5	40.1	62.5	66	49.2	BSC
10	IMRT	55	none	none	24.9	516.1	1355.4	38.1	31.5	57.4	52.5	32.2	BEV/IRI
11	VMAT	60	TMZ	none	89.3	349.5	1458.7	24	36.8	60.4	63.9	39.2	BSC
12	VMAT	50	BEV	BEV	303.9	888.5	1406.5	63.2	36.4	51.2	79.7	14.9	BSC
13	VMAT	60	TMZ	none	16.9	333.2	1285.2	25.9	32.5	61.8	56.1	34.2	BSC
14	VMAT	60	TMZ	TMZ	3.6	290	1241.4	23.4	30.8	62	40.8	29.2	ReRT + TMZ
15	VMAT	60	TMZ	CeTeG	13.3	192.6	1451.2	13.3	27.9	61.5	34.3	23.7	NA
16	3D CRT	60	TMZ	none	23.7	373.25	1175.1	31.8	39.7	61.7	61.5	48.9	BSC
17	VMAT	60	CeTeG	CeTeG	27.1	203.4	1599.6	12.7	25.3	61.7	33	21	TMZ ReRT GLIAA
18	VMAT	60	TMZ	TMZ	39.6	400.8	1187.1	33.8	36.1	61.8	56.5	39.6	BSC
19	VMAT	60	TMZ	TMZ	27.4	375.6	1410.8	26.6	31.9	62.1	44.6	33.6	BSC
20	VMAT	60	none	none	12.5	161.6	1342.1	12	31.4	61.4	42.1	21.2	BSC
Median	NA	60	NA	NA	26	425.7	1397.65	32.8	37	61.5	59.9	40.7	NA

### Survival

**Fig. 2:**
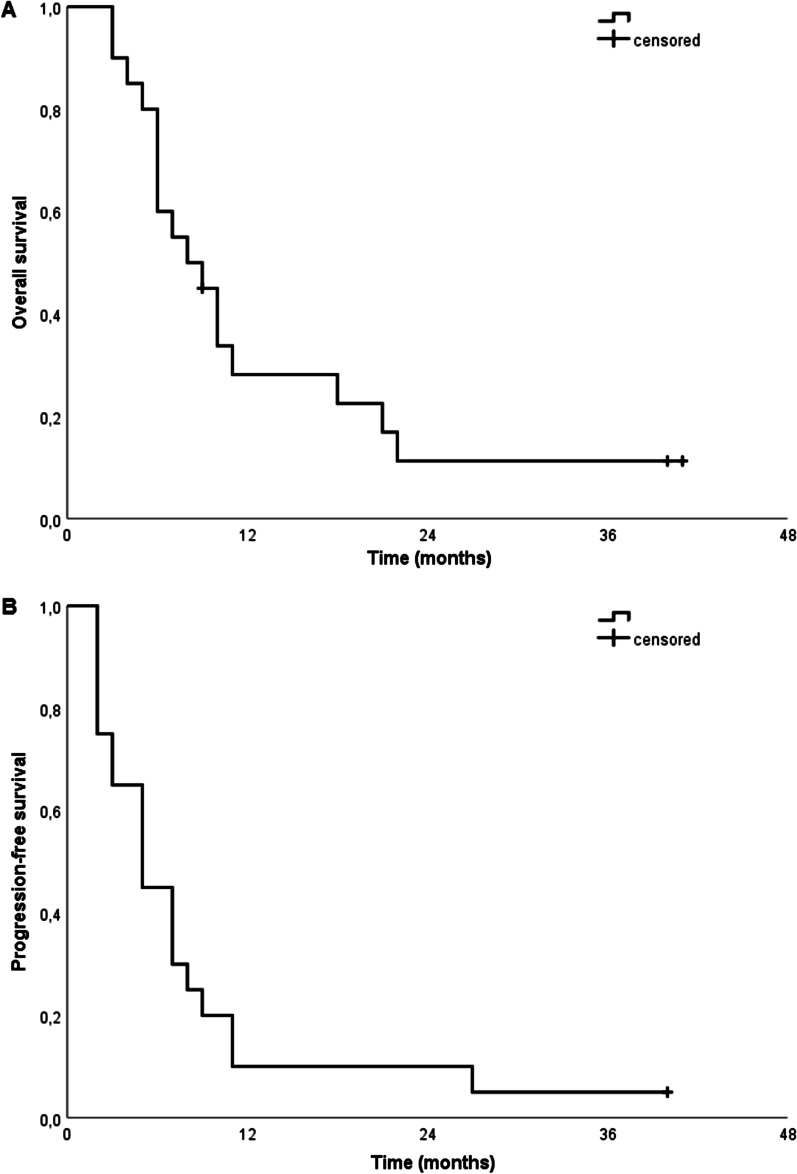
Overall and progression-free survival. Overall survival after diagnosis of multifocal high-grade glioma and progression-free survival after initiation of radiotherapy

Median survival after diagnosis was eight months (95% KI 3.6–12.4 months) and median progression-free survival after initiation of RT five months (95% CI 2.8–7.2 months) (Fig. 2). Median survival and progression-free survival was not significantly different between patients with PTV volumes greater than 425.7 cm^3^ and patients with smaller PTV volumes (6 vs. 10 months, *p* = 0.24; 5 vs. 5 months, *p* = 0.298) or between patients with methylated and unmethylated MGMT promotors (7 vs. 9 months, *p* = 0.615; 5 vs. 7 months, *p* = 0.804). A trend towards longer median survival and progression-free survival was seen for patients with KPS > 80 prior to initiation of RT (9 vs. 6 months, *p* = 0.076; 5 vs. 3 months, *p* = 0.1) as well as for patients with KPS ≥ 80 at the end of RT (10 vs. 6 months, *p* = 0.025, 7 vs. 5 months, *p* = 0.154).

### Adverse events

Grade 2 toxicities were detected in eight cases including alopecia, cushingoid symptoms, fatigue, hyperglycaemia, intracranial haemorrhage, platelet count decrease, thromboembolic events, and vomiting. Grade 3 toxicities were detected in four cases with three cases of increasing cerebal edema and one case of febrile neutropenia and one case of seizure. One grade 4 toxicities with severe white blood cell count decrease related to concomitant chemotherapy was observed. Subclassification of these adverse events according to CTCAE v. 5.0 is shown in Table 3. For grade 2 toxicities, V30, V45 and the D2 values were above or equal to median in 4/8, 3/8 and 4/8 cases, respectively. For grade 3 and 4 toxicities V30, V45 and the D2 values were above or equal to median in 0/4, 0/4 and 2/4 cases, respectively.

**Table 3:** Adverse events

Pat	Grade 1 toxicities	Grade 2 toxicities	Grade 3 toxicities	Grade 4 toxicities
1	Fatigue Headache	–	Seizure	–
2	Fatigue	–	–	–
3	Alopecia ALT and AST increase Fatigue	–	–	–
4	Dermatitis	–	–	–
5	Alopecia Dermatitis Fatigue	Thromboembolic event	–	–
6	Alopecia Fatigue Headache	–	–	–
7	Blurred vision Cognitive disturbance Dizziness	Cushingoid Platelet count decrease Vomiting	–	–
8	Alopecia Dysphasia Headache	Intracranial hemorrhage	–	–
9	Fatigue	–	–	–
10	Alopecia Fatigue Headache Trigeminal nerve disorder	–	–	–
11	Alopecia Blurred vision Dysphasia Fatigue Headache Vomiting	–	–	–
12	Fatigue Hypersomnia Memory impairment	Cushingoid Hyperglycemia	–	–
13	Dermatitis Fatigue	Platelet count decrease	Cerebral edema	Febrile neutropenia White blood cell decrease
14	Fatigue	Alopecia Thromboembolic event		
15	Fatigue	–	–	–
16	Alopecia Fatigue Headache	–	Cerebral edema	–
17	ALT and AST increase	–		–
18	Alopecia Fatigue	Platelet count decrease	Cerebral edema	–
19	Alopecia Fatigue	–	–	–
20	-	Alopecia Fatigue	–	–

## Discussion

There are no definitive standards for the diagnosis and treatment of patients with multifocal high-grade gliomas. The current WHO classification of tumours of the central nervous system does not differentiate between multifocal and unifocal high-grade gliomas [2], although a number of studies on the histopathology of multifocal high-grade gliomas postulate, that distinct histopathological differences are observed [3–6]. Current guideline recommendations do not address multifocal high-grade glioma separately from the unifocal presentation of the disease [10]. Since few investigator initiated trials on RT treatment exist [22], it is no surprise that there is only very limited evidence for the radiotherapeutic treatment of patients with multifocal glioblastoma.

For a better understanding of the multifocal form of high-grade glioma, we examined a set of 20 unselected multifocal high-grade glioma cases, treated with conventionally fractionated, limited field RT with modern techniques including 3D conformal, IMRT and VMAT with or without concomitant chemotherapy. The focus of this case series was the assessment of the treatment outcomes in terms of progression-free and overall survival and treatment related adverse events for conventionally fractionated RT limited-field radiation treatment regimens. The VMAT technique, which enables more advanced dose modulation in glioblastoma treatment planning such as hippocampus [23, 24] and normal tissue sparing [25], was applied in almost half of the cohort, i.e. in nine of the 20 cases.

Despite an aggressive treatment approach, progression-free and overall survival in the present cohort were markedly shorter than in comparable high-grade glioma cohorts with predominantly unifocal tumours treated with RT and concomitant daily temozolomide, with a median overall survival of up to 15.7 months [7, 8, 26]. When comparing the present cohort to other cohorts treated with radiotherapy and daily administration of temozolomide without prior surgical resection, overall survival times are comparable with 7 vs. 9.2 months, respectively [15]. In previous RT case series of multifocal high-grade glioma patients treated with modern treatment techniques, the overall survival was comparable to our case series, with reported median overall survival times in the range between 8.2 months [6], 8.7 months [13] and 11.5 months [16].

Shortcomings of the study are its retrospective nature, the limited number of patients and also the heterogeneity of the treatment regimens used. Due to the rarity of the disease and the long period of time during which patients were included, different radiation doses and techniques were used. The predominant irradiation technique applied until 2014 was 3D conformal RT, followed by IMRT until 2016 and VMAT from 2017 onwards. Chemotherapy regimens also changed over time. For example, the combination of temozolomide and lomustine according to the CeTeG/NOA-09 trial protocol [18] was introduced following the encouraging results presented at the SNO annual meeting in 2017. In contrast, the NOA-05 trial including 35 patients with gliomatosis cerebri treated with primary chemotherapy with procarbazine and lomustine showed remarkable median progression-free and overall survival times of 14 months and 30 months, respectively. The phase 2 setting of this chemotherapy study is of course different to this unselected real life cohort, therefore the results are not fully comparable. Furthermore, it has also to be taken into account that one third of the patients of the NOA-05 trial received radiotherapy after the primary chemotherapy [27].

One of the main reasons for the poor overall survival of multifocal high-grade glioma patients could be the reduced performance status of the patients, which was also evident in the present cohort with a median KPS of 85 prior to the initiation of RT and 80 at the end of RT, respectively. A KPS above median prior to and at the end of RT, respectively, showed a trend towards longer survival in this series, even though statistical evaluations have to be looked at with caution due to the small number of cases.

Histopathologically, it has been discussed that the higher phenotypic aggressiveness of multifocal glioma itself might explain the poorest survival of all glioma subtypes [6, 28]. The risk of refractory edema caused by large tumour infiltration and large RT treatment volumes with the prolonged need for dexamethasone after the completion of RT can also be discussed as a reason for poorer overall survival in patients with multifocal tumours. Interestingly, the three cases with grade 3 edema were cases with PTV volume below or in the range of the median, so the PTV volume by itself may not be the determining factor for the occurrence of edema after radiotherapy.

Whole brain radiotherapy (WBRT), which was the standard of care prior to the introduction of 3D conformal RT had considerable worse treatment outcomes with reported median overall survival times of only 3.7 months [13]. However, a recently reported monocentric case series of WBRT with concomitant and adjuvant TMZ chemotherapy in newly diagnosed multifocal glioblastoma patients reported a comparable overall survival of 10 months in median. Reported toxicities of this WBRT series were comparable to the limited field RT of this series with three grade 3 toxicities and one grade 4 toxicity [17].

A recent large-scale study initiating a nomogram for survival prediction of glioblastoma patients and a subsequent validation study have shown that a low KPS and lack of gross total resection, as present in the current case series, are significantly correlated with poorer overall survival [29, 30]. Of note, multifocality itself was not included in this nomogram, possibly because of the rarity of this condition [29, 30]. In contrast, radiomics approaches, which are increasingly used for prognostic assessment of glioblastoma patients, multifocality is used as one of the main imaging features [31–33].

Large database studies have shown that concomitant systemic treatment with temozolomide has a benefit specifically in patients who could not undergo a surgical resection of the tumour, in both unifocal and multifocal growth patterns [1, 15]. Nevertheless, further information about toxicities related to concomitant temozolomide in multifocal high-grade glioma patients could not be determined in these studies, as it was not documented in the databases. In our series, concomitant chemotherapy with temozolomide was applied in 17 patients with mostly acceptable toxicity, but one patient developed a grade 4 leukopenia.

In our unselected limited field RT cohort, adverse events were manageable despite the high percentages of irradiated brain volume of up to 63.2 percent. Only one case with a grade 4 toxicity was detected, which was not related to radiation but to concomitant chemotherapy, i.e. a severe decrease in white blood cells associated resulting in neutropenic fever. Grade 3 toxicities consisted of one case with new-onset seizure possibly related to radiation treatment and increasing cerebral edema in three cases, which did not appear to be related to the size of the PTV volume or above median values of V30, V45 and D2 of the brain.

## Conclusion

In this case series, multifocal high-grade glioma could be treated safely with conventionally fractionated RT with concomitant and adjuvant TMZ chemotherapy. Prospective studies are warranted to select the best treatment regimen for multifocal high-grade glioma patients to improve the oncological outcome.

## Data Availability

All data generated or analysed during this study are included in this published article.
